# Periostin is associated with prognosis and immune cell infiltration in pancreatic adenocarcinoma based on integrated bioinformatics analysis

**DOI:** 10.1002/cnr2.1990

**Published:** 2024-02-22

**Authors:** Yijun Chen, Fengyu Zhang, Bolin Zhang, Bogusz Trojanowicz, Monika Hämmerle, Jörg Kleeff, Yoshiaki Sunami

**Affiliations:** ^1^ Department of Visceral, Vascular and Endocrine Surgery Martin‐Luther‐University Halle‐Wittenberg, University Medical Center Halle Halle (Saale) Germany; ^2^ School of Biomedical Engineering and Technology Tianjin Medical University Tianjin China; ^3^ Institute of Pathology, Martin‐Luther‐University Halle‐Wittenberg University Medical Center Halle Halle (Saale) Germany

**Keywords:** bioinformatics, cancer‐associated fibroblasts, pancreatic cancer, periostin, tumor‐associated macrophages

## Abstract

**Background:**

Pancreatic cancer is one of the most aggressive human malignancies. Previous research has shown that periostin (POSTN) promotes pancreatic cancer cell proliferation, migration, and invasion. Further, POSTN is involved in tumor microenvironment remodeling during tumor progression. However, the relationship between POSTN expression, immune cell infiltration, and the efficacy of immunotherapy in pancreatic cancer is unclear.

**Methods:**

We conducted a comprehensive evaluation of POSTN differential expression, examining mRNA and protein levels. To gather data, we utilized various databases including gene expression profiling interactive analysis 2 (GEPIA2), gene expression omnibus (GEO), and the human protein atlas (HPA). To investigate the correlation between *POSTN* expression and clinical characteristics, we analyzed data from the Kaplan–Meier plotter database and clinical data sourced from the cancer genome atlas (TCGA). Furthermore, we performed gene ontology (GO) analysis, Kyoto encyclopedia of genes and genomes (KEGG) pathway analysis, and gene set enrichment analysis (GSEA). Additionally, we explored the relationship between *POSTN* expression and immune cell infiltration, as well as the immunophenoscore (IPS), by leveraging the cancer immunome atlas (TCIA) database. Lastly, we examined the tumor mutational burden (TMB) in pancreatic cancer in relation to *POSTN* expression.

**Results:**

When compared with healthy pancreatic tissues, pancreatic cancer tissues displayed significantly higher levels of POSTN, which was indicative of a worse prognosis. *POSTN* expression was closely associated with extracellular matrix (ECM) organization, ECM‐receptor interaction, and focal adhesion by GO, KEGG pathway, and GSEA analyses. Higher expression of *POSTN* was associated with increased infiltration of M2 macrophages. Additionally, increased IPS was linked to lower *POSTN* expression. IPS scores for CTLA4, PD‐1/PDL1, and CTLA4/PD‐1/PDL1 immune checkpoint inhibitors were also higher in the *POSTN*‐low expression group, suggesting that lower expression of *POSTN* is associated with a better outcome with checkpoint inhibitor treatment.

**Conclusion:**

POSTN is related to pancreatic cancer prognosis, and may influence immune cell infiltration. High expression of *POSTN* is predicted to correlate with lower sensitivity to immunotherapy with checkpoint inhibitors in pancreatic cancer.

## 1 INTRODUCTION

Pancreatic cancer is the fourth most common cause of cancer‐associated mortality in the United States, and the 5‐year relative survival rate is still only about 12%.^1^ By 2030, it is expected to become the second deadliest cancer in the United States.^2^ Pancreatic cancer poses a major clinical challenge, both because of its late diagnosis and its remarkable resistance to established therapeutic options such as chemotherapy and radiotherapy.^3^, ^4^ Pancreatic cancer is at least in part driven by somatic mutations in oncogenes such as *KRAS* and tumor suppressor genes like *TP53*, *CDKN2A*, and *SMAD4* and other molecular alterations. Increasing evidence shows that interactions with stromal cells in the tumor microenvironment play a critical role in pancreatic tumorigenesis.^3^, ^4^, ^5^

Cancer‐associated fibroblasts (CAFs), which are partly differentiated from pancreatic stellate cells (PSCs) and endothelial cells, are key sources for inflammatory cytokines, chemokines, growth factors, and extracellular matrix (ECM) components in the tumor microenvironment.^3^ Besides CAFs, the ECM components contain tumor‐associated macrophages (TAMs) and immunosuppressive or exhausted T cells, which are also critically involved in tumor progression.^6^, ^7^ Although the critical role of the immune system in safeguarding the body against cancer development has been widely recognized, several studies demonstrate that immune suppression acts as a promoting factor in pancreatic cancer.^8^ CAF and TAM emerge as key players in orchestrating tumor‐promoting inflammation and immunotherapy resistance.^9^

Periostin (encoded by the *POSTN* gene) is an ECM protein secreted by CAFs and involved in tumor microenvironment remodeling during tumor progression.^10^ Previous studies have demonstrated that POSTN expression levels may promote pancreatic cancer proliferation, migration, and invasion.^11^, ^12^ However, it remains largely unclear whether and how POSTN expression is associated with the mutational status and immune cell infiltration.

In the current study, we observed that higher POSTN expression is associated with a poorer prognosis in pancreatic cancer, leading to the infiltration of immune‐suppressive cells, especially M2 macrophages. High POSTN expression is predicted to result in reduced sensitivity to immunotherapies involving checkpoint inhibitors in pancreatic cancer.

## 2 MATERIALS AND METHODS

### 2.1 GEPIA2 database analysis

The gene expression profiling interactive analysis 2 (GEPIA2) database (http://gepia2.cancer-pku.cn/#index),^13^ which includes gene expression data from the cancer genome atlas (TCGA) (https://portal.gdc.cancer.gov/) and GTEx (https://gtexportal.org/) databases, was used to explore the expression of *POSTN* in different cancer types and normal tissues.

### 2.2 TCGA and UCSC XENA data acquisition and analysis

TCGA‐pancreatic adenocarcinoma (PAAD) RNA‐seq expression data in transcript per million (TPM) format and the corresponding clinical information were obtained from the TCGA database, which contains 178 pancreatic cancer samples. Normal samples and cystic, mucinous, and serous neoplasms were excluded unless stated otherwise. GTEx‐PAAD RNA‐seq expression data (TPM format) were obtained from the UCSC XENA database (http://xena.ucsc.edu/), which comprised 167 normal pancreas samples. The samples were employed to investigate variations in *POSTN* expression through the application of an unpaired *t*‐test or a nonparametric test. Box plots were utilized to visually depict the associations between *POSTN* expression and clinical characteristics, including TNM stage and grade, using the TCGA dataset.

### 2.3 GEO data acquisition and analysis

Pancreatic cancer microarray data from gene expression omnibus (GEO) databases (GSE15471: 39 tumor samples and 39 juxta‐tumoral tissue samples, GSE28735: 45 tumor samples and 45 juxta‐tumoral tissue samples, GSE62452: 69 tumor samples and 61 juxta‐tumoral tissue samples) (https://www.ncbi.nlm.nih.gov/geo/) were used to analyze the expression of *POSTN*.

### 2.4 Human protein atlas database analysis

The human protein atlas (HPA) version 22.0 (https://www.proteinatlas.org/) was used to assess POSTN expression in normal pancreas and pancreatic cancer tissues at the protein level by immunohistochemistry.^14^

### 2.5 Human subjects

Human pancreatic cancer samples were obtained from the University Medical Center Halle, Martin‐Luther‐University Halle‐Wittenberg, Germany. The study on human material was approved by the institutional ethics board of the medical faculty of the Martin‐Luther‐University Halle‐Wittenberg and designated in accordance with the Declaration of Helsinki with the approval number 2019‐037. Written consent was obtained from all patients.

### 2.6 Immunohistochemistry

Paraffin blocks of human pancreatic cancer samples were cut into 4 μm sections for immunohistochemical staining. The slides were deparaffinized and microwaved in 5.9 mM citrate buffer (pH 6.0) for antigen retrieval. Afterwards, the slides were blocked with 1% bovine serum albumin in phosphate buffered saline. The sections were subsequently incubated with anti‐periostin antibody (Abcam ab14041, 1:500) or CD163 monoclonal antibody (Invitrogen 10D6, 1:50) and corresponding secondary antibody (anti‐rabbit Dako K0675). Finally, signals were detected by using Dako DAB+ chromogen kit (Dako K3468, 1:500).

### 2.7 Kaplan–Meier plotter database analysis for pancreatic cancer patients

Kaplan–Meier Plotter database (http://kmplot.com/analysis) was used to investigate the correlation between *POSTN* expression and the prognosis of pancreatic cancer.

### 2.8 Kaplan–Meier survival analysis for the classical and basal‐like pancreatic cancer subtypes

We procured the TCGA pancreatic cancer data via the HPA database, which incorporates individual patient survival information (https://www.proteinatlas.org/ENSG00000133110‐POSTN/pathology/pancreatic+cancer). Subsequently, we conducted an examination of the Pancreatic Expression Database (https://pancreasexpression.org/analytics/cohort/tcga/),^15^ which provides details regarding pancreatic cancer subtypes. We defined patients in *POSTN*‐high and ‐low groups according to Kaplan–Meier plotter database. Kaplan–Meier survival analyses with log‐rank (Mantel–Cox) test were conducted for classical and basal‐like pancreatic cancer subtypes, utilizing GraphPad Prism software, version 8.4.3 (GraphPad software, Boston, MA).

### 2.9 Tumor mutational burden analysis

We utilized the R package “Maftools” to generate visual representations of mutation profiles in pancreatic cancer cases categorized into high and low levels of *POSTN* expression. The “somaticinteractions” function was employed to assess the correlation of the top 20 mutated genes. To determine the tumor mutational burden (TMB), we calculated the ratio of the total variant count, encompassing base substitutions, insertions, deletions, or insertions across bases, to the total exon length. Subsequently, we conducted the Wilcoxson test to analyze the disparity in TMB between the subgroups with high and low *POSTN* expression. TMB was computed as the total variant count divided by the total exon length. The correlation between *POSTN* gene expression and TMB was calculated by using the Spearman correlation test method and visualized by R package “ggplot2.”

### 2.10 Analysis differentially expressed genes

According to the median *POSTN* expression, the RNA‐seq TPM data of pancreatic cancer from the TCGA database were divided into two groups: *POSTN*‐high and *POSTN*‐low expression groups. The differentially expressed genes (DEGs) were analyzed, and volcano diagrams were drawn by using R package “Deseq2,” which normalizes un‐normalized data. The screening criteria were |logFC| > 1, *p* < .05. R package “clusterprofiler” was used for gene ontology (GO) and Kyoto encyclopedia of genes and genomes (KEGG) analysis. For analyzing *POSTN* co‐expression immune genes, 2483 immune‐related genes were downloaded from the ImmPort database (http://www.immport.org). The immune‐related genes were divided into 17 categories such as antigen processing and presentation, chemokines, tumor necrosis factor family receptors. Immune genes co‐expressed with *POSTN* were screened by Pearson correlation analysis, the screening criteria was |*r*| > 0, adjusted *p* < .05.

### 2.11 Gene set enrichment analysis

We performed gene set enrichment analysis (GSEA) using the “clusterprofiler” R package to identify significant functional and pathway differences between the groups with high‐ and low‐*POSTN* expression. Each analysis was iterated 1000 times. For statistical significance, a function or pathway term was considered significant if it had an adjusted *p*‐value <.05 and false discovery rate (FDR) < 0.25.

### 2.12 Calculation of the TME's immune score

Estimation of stromal and immune cells in malignant tumor tissues using expression data (ESTIMATE) algorithm was used to predict the amount of infiltrating immune cells and stromal cells in tumor tissue by calculating the immune score and matrix score of each sample.

### 2.13 Acquisition and analysis of single nucleus RNA‐seq data

We utilized a human, treatment‐naïve single nucleus RNA‐seq dataset consisting of 88 031 cells on the Single Cell Portal (https://singlecell.broadinstitute.org/single_cell/study/SCP1089/human‐treatment‐naive‐pdac‐snuc‐seq), to investigate the expression of *POSTN* across various cell types in pancreatic cancer.

### 2.14 Immune infiltration analysis

We investigated the correlation between *POSTN* expression and immune cell infiltration using two different tools within the TCGA_PAAD cohort. Initially, we utilized the “CIBERSORT” algorithm to determine the proportions of 22 immune cell types in a cohort of 178 patients. A significance threshold of *p* < .05 was applied to identify samples where the inferred fractions of immune cell populations provided by CIBERSORT. Subsequently, we employed the Wilcoxon test to assess differences in immune cell fractions between the *POSTN*‐high and *POSTN*‐low groups. Furthermore, we validated the positive correlation between *POSTN* expression and the expression of the M2 macrophage marker using the GEPIA2 platform.

### 2.15 The relationship between *POSTN* expression, Tumor Immune Dysfunction and Exclusion score, and immunophenoscore

Tumor Immune Dysfunction and Exclusion (TIDE) estimates two different methods of tumor immune evasion, including dysfunction of tumor‐infiltrating cytotoxic T cells (CTLs), and exclusion of CTLs by immunosuppressive agents, using a collection of gene expression indicators. TIDE score simulates the immune escape mechanisms of tumor immune dysfunction and exclusion to predict the effect of immunotherapy. The higher the TIDE score, the smaller the effect of immunotherapy. Immunophenoscore (IPS) score and somatic mutation data from the cancer immunome atlas (TCIA) database (https://tcia.at/) and the TCGA database were downloaded and divided into two groups according to the expression of *POSTN*. IPS was calculated based on the sum of the weighted average of four categories, namely, effector cells, suppressor cells, major histocompatibility complex‐related molecules, and checkpoint molecules or immunomodulators. The detailed calculation schema has been described previously.^16^

## 3 RESULTS

### 3.1 High POSTN expression is associated with poor survival in pancreatic cancer patients

**FIGURE 1:**
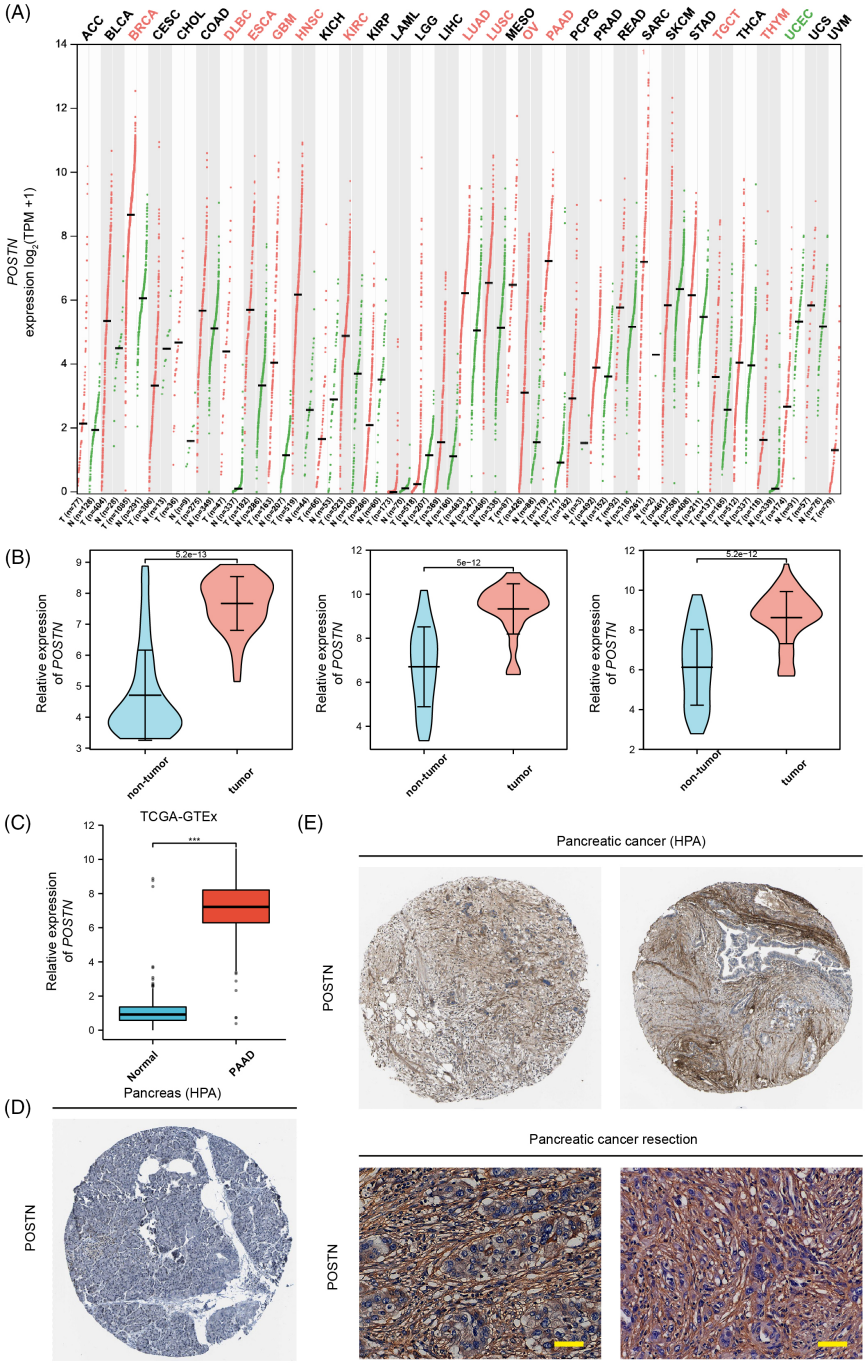
The expression of *POSTN* is elevated in several types of cancer including pancreatic cancer. (A) *POSTN* expression at the mRNA level in different cancers and paired normal tissues. (B) *POSTN* mRNA expression data from three pancreatic cancer GEO series. (C) *POSTN* expression at the mRNA level in pancreatic cancer and normal pancreas tissues in the cancer genome atlas (TCGA) and GTEx (via XENA) databases respectively. (D) Representative POSTN immunohistochemical staining of normal pancreas from the human protein atlas (HPA) database (Patient ID 3320, low staining, moderate intensity, and <25% quantity in exocrine glandular cells, low staining, weak intensity, and 75%–25% quantity in pancreatic endocrine cells, http://www.proteinatlas.org/ENSG00000133110‐POSTN/tissue/pancreas#img). (E) Representative POSTN staining of pancreatic cancer from the HPA database (upper left panel: Patient ID 3004, low staining, weak intensity, and >75% quantity in tumor. Upper right panel: ID 729, medium staining, moderate intensity, 75%–25% quantity in cancer. http://www.proteinatlas.org/ENSG00000133110‐POSTN/pathology/pancreatic+cancer#img) as well as pancreatic cancer patient specimens (lower panel) (bar: 50 μm). TPM, transcript per million.

To evaluate the differential expression of *POSTN* in cancer tissues at mRNA levels, we first analyzed the GEPIA2 program. We found that *POSTN* gene was up‐regulated in several cancer types as compared to the respective healthy tissues such as invasive breast carcinoma, diffuse large B‐cell lymphoma, esophageal carcinoma, glioblastoma multiforme, head and neck squamous cell carcinoma, kidney clear cell carcinoma, lung adenocarcinoma, lung squamous cell carcinoma, ovarian serous cystadenocarcinoma, testicular germ cell tumors, thymoma, and in PAAD (Figure 1A). Increased expression of *POSTN* in PAAD tissues as compared to normal pancreatic tissues was further confirmed by analyzing gene expression data of *POSTN* from the TCGA and UCSC XENA databases, as well as in different GEO datasets (GSE15471, GSE28735, and GSE62452) (Figure 1B,C). Further, we analyzed representative immunohistochemical samples from the HPA database as well as from pancreatic cancer patients at our institution (UKH). We observed strong stromal POSTN staining in pancreatic cancer tissues (UKH) but not in normal samples (Figure 1D,E).

**FIGURE 2:**
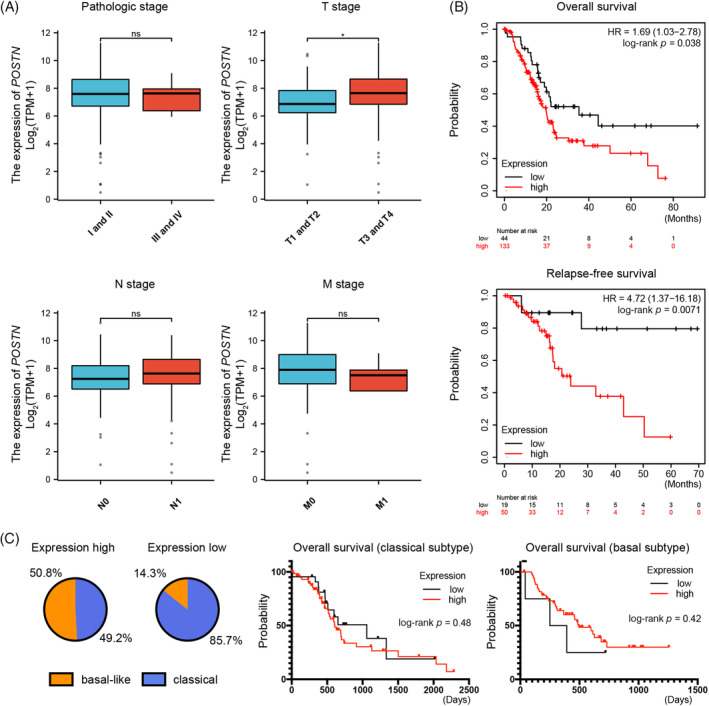
*POSTN* expression is associated with shorter overall as well as relapse‐free survival in pancreatic cancer. (A) Association between *POSTN* expression and pathologic/TNM stages (American Joint Committee on Cancer). (B) Overall survival and relapse‐free survival for the *POSTN*‐high and ‐low expressing groups in pancreatic cancer patients from the Kaplan–Meier plotter database. (C) Parts of whole diagrams for the high‐ and low *POSTN* expression groups and subtype‐specific Kaplan–Meier overall survival data. TPM, transcript per million.

The characteristics of pancreatic cancer patients including gender, age, histological grade, pathological stage, and TNM stages (American Joint Committee on Cancer) were extracted from the TCGA database. According to median *POSTN* expression, we divided 178 pancreatic cancer samples into groups of *POSTN*‐high and *POSTN*‐low expression. There was no correlation between *POSTN* expression and gender, age, grade, or TNM stage (Table 1). There was also no correlation between pathologic stage I and II, and III and IV (*p* = .41), yet increased *POSTN* expression was observed in T3 and T4 tumors as compared to T1 and T2 tumors (*p* < .05) (Figure 2A). We next analyzed the Kaplan–Meier plotter database, which revealed that high expression of *POSTN* was associated with shorter overall (OS) (HR = 1.69, 95% CI: 1.03–2.78, *p* = .038) as well as relapse‐free survival (RFS) (HR = 4.72, 95% CI: 1.37–16.18, *p* = .0071) in pancreatic cancer patients (Figure 2B). Taken together, *POSTN* expression was higher in pancreatic cancer patients and was associated with shorter OS and RFS.

**TABLE 1:** *POSTN* expression and clinicopathological features of pancreatic cancer patients.

	POSTN‐low expression	POSTN‐high expression	*p* value
*n*	89	89	
Gender, *n* (%)			.880
Female	41 (23.0)	39 (21.9)	
Male	48 (27.0)	50 (28.1)	
Age, *n* (%)			.230
≤65	42 (23.6)	51 (28.7)	
>65	47 (26.4)	38 (21.3)	
Histologic grade, *n* (%)			.207
G1	20 (11.4)	11 (6.2)	
G2	46 (26.1)	49 (27.8)	
G3	20 (11.4)	28 (15.9)	
G4	1 (0.6)	1 (0.6)	
Not known	2	0	
Pathologic stage, *n* (%)			.413
Stage I	14 (8.0)	7 (4.0)	
Stage II	71 (40.6)	75 (42.9)	
Stage III	1 (0.6)	2 (1.1)	
Stage IV	3 (1.7)	2 (1.1)	
Not known	0	3	
T stage, *n* (%)			.139
T1	4 (2.3)	3 (1.7)	
T2	17 (9.7)	7 (4.0)	
T3	67 (38.1)	75 (42.6)	
T4	1 (0.6)	2 (1.1)	
Not known	0	2	
N stage, *n* (%)			.649
N0	27 (15.6)	23 (13.3)	
N1	60 (34.7)	63 (36.4)	
Not known	2	3	
M stage, *n* (%)			.645
M0	32 (38.1)	47 (56.0)	
M1	3 (3.6)	2 (2.4)	
Not known	54	40	
Age, median (IQR)	67 (61, 74)	64 (55, 73)	.054

Abbreviation: IQR, interquartile range.

It has been demonstrated that there are two predominant molecular subtypes of pancreatic cancer, namely basal‐like and classical. The basal‐like subtype of pancreatic cancer is associated with poor survival of patients.^17^ To determine whether expression of *POSTN* is higher predominantly in basal‐like subtype, we first obtained TCGA pancreatic cancer data via the HPA database, which included survival information for 176 individual patients. Subsequently, we conducted an analysis of the Pancreatic Expression Database, which comprises data from 179 pancreatic cancer patients, providing subtype information for 148 cases. In the *POSTN*‐high expression group, we observed 61 cases (50.8%) classified as basal‐like, and 59 cases (49.2%) as classical subtype. In the *POSTN*‐low expression group, only 4 cases (14.3%) were classified as basal‐like subtype, while 24 cases (85.7%) were classified as classical subtype. However, Kaplan–Meier overall survival analyses did not reveal any significant differences between *POSTN*‐high and *POSTN*‐low expression groups for both classical (*p* = .48) and basal‐like (*p* = .42) subtypes (Figure 2C).

### 3.2 POSTN expression is not associated with the mutational status

**FIGURE 3:**
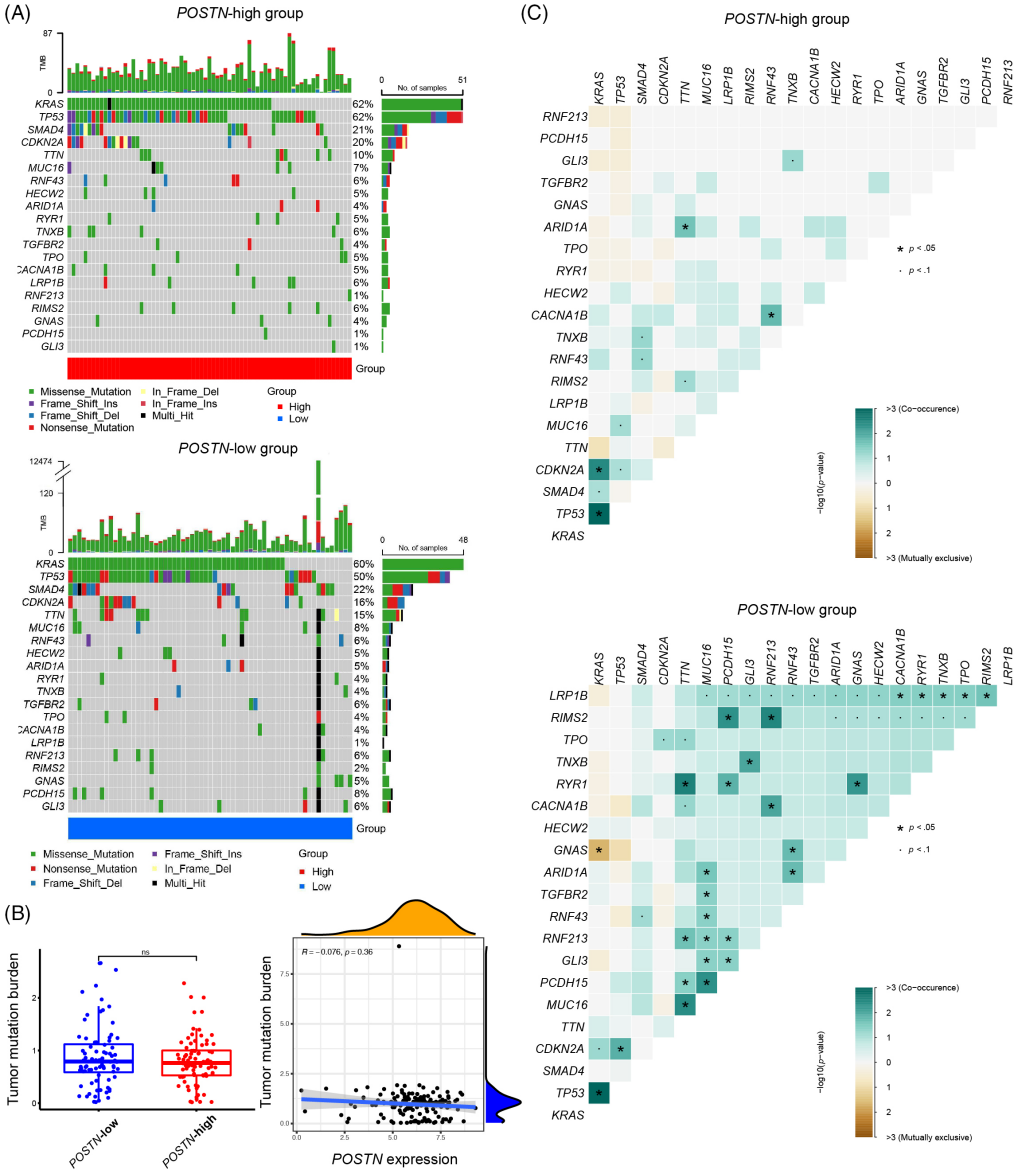
No significant difference on tumor mutation burden between *POSTN*‐high expression and low expression groups, but co‐occurrence of gene mutations are more evident in the *POSTN*‐low expression group. (A) Waterfall plot of mutation information of top 20 genes, (B) tumor mutation burden for *POSTN*‐high and *POSTN*‐low expression groups (left panel) as well as correlation between tumor mutation burden and *POSTN* expression (right panel), and (C) correlation between top 20 mutated genes.

On the molecular level, it has been widely recognized that malignant transformation involves mutation of several oncogenes and tumor suppressor genes such as *KRAS* and *TP53* in pancreatic cells.^5^ To analyze, whether expression of *POSTN* is associated with the somatic mutation status of oncogenes and tumor suppressor genes, we compared the TMB in *POSTN*‐high and ‐low expression samples. Complete somatic mutation data were available for 162 pancreatic cancer patients, and we analyzed the 20 most frequently mutated genes **(**Figure 3A). In the high expression group, 71 cases from 82 patients (86.6%) were detected with somatic mutations in at least one of the top 20 genes and 63 from 80 cases (78.8%) for the low expression group (Figure 3A). In both groups, *KRAS*, *TP53*, *SMAD4*, and *CDKN2A* were consistently identified as the four most frequently mutated genes (Figure 3A). Patients with high *POSTN* expression had similar TMB compared with those with low *POSTN* expression. We further analyzed correlation between TMB and *POSTN* expression and observed that *POSTN* expression is not associated with the TMB (Figure 3B). Further, we analyzed the correlation for these top 20 mutated genes. Interestingly, the correlation for the mutated genes was frequently observed in the low *POSTN* expression group, but rare in the high *POSTN* expression group (Figure 3C). These data suggest that *POSTN* expression is not associated with mutational status, but high *POSTN* expression may reduce the significance of mutual association between different gene mutations.

### 3.3 Expression of *POSTN* is positively associated with immune‐related gene expression

**FIGURE 4:**
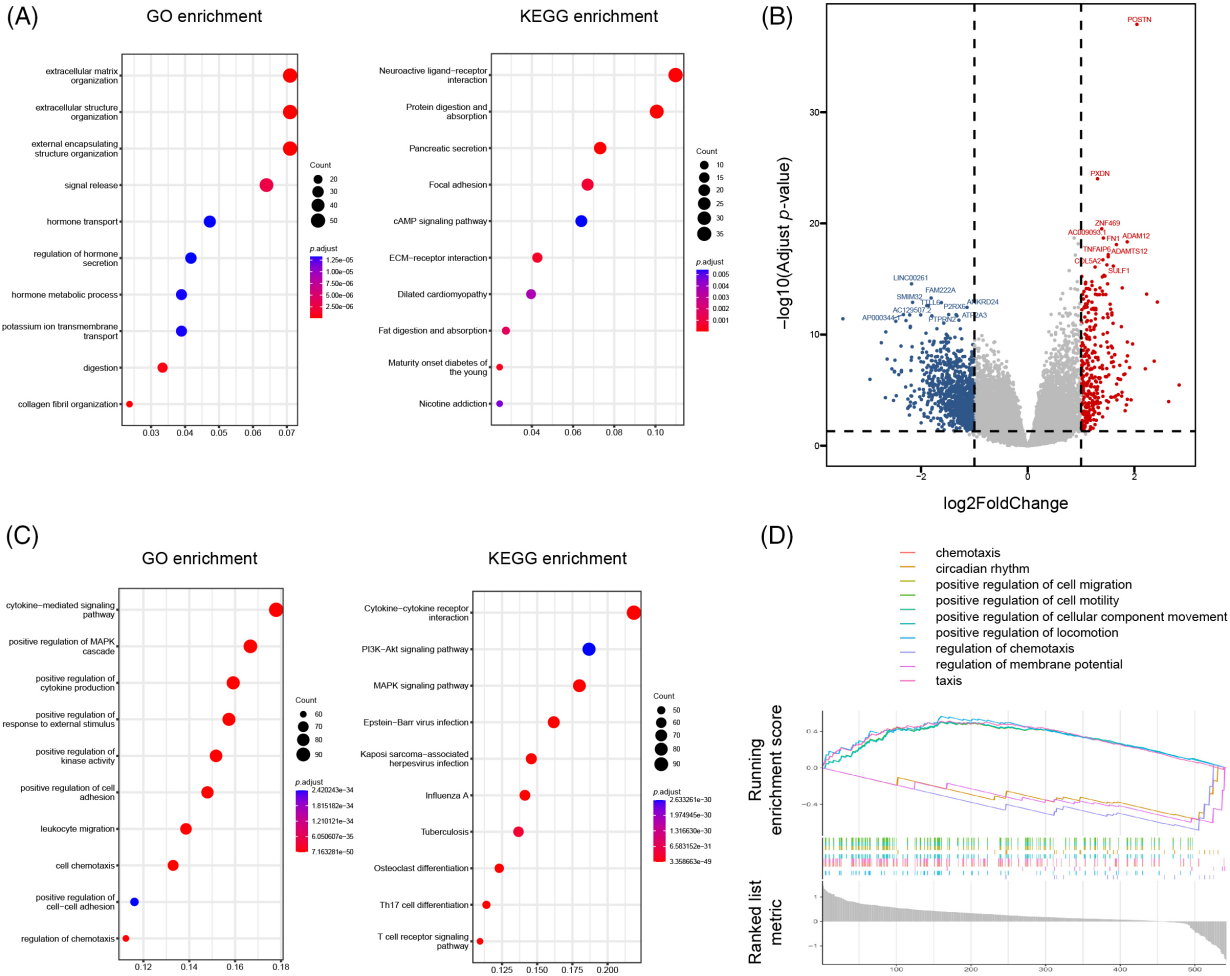
Enrichment analysis of *POSTN*‐associated differentially expressed genes. (A) Differentially expressed genes in *POSTN*‐high and low expression groups, (B) gene ontology (GO) and Kyoto encyclopedia of genes and genomes (KEGG) enrichment analysis of *POSTN*‐associated differentially expressed genes, (C) GO and KEGG enrichment analysis of *POSTN*‐associated immune‐related differentially expressed genes, and (D) gene set enrichment analysis enrichment analysis of differentially expressed immune‐related genes.

To further clarify the role of periostin in pancreatic cancer, we screened DEGs depending on *POSTN* expression levels. In the high POSTN expression group compared to the low POSTN expression group, we identified 337 up‐regulated genes and 1044 down‐regulated genes (|logFC| > 1, *p* < .05). Subsequently, we performed GO and KEGG analyses to further investigate these DEGs. The data showed that *POSTN* expression was closely connected with the ECM organization, extracellular structure organization, external encapsulating structure organization, focal adhesion, and ECM‐receptor interaction (Figure 4A). Interestingly, we identified peroxidasin (PXDN, also known as vascular peroxidase‐1), whose expression was positively correlated with *POSTN* (Figure 4B). A recent bioinformatics study highlighted the involvement of PXDN in the infiltration of fibroblasts and various immune cells, suggesting its potential as a promising target for tumor immunotherapy.^18^ CAFs and the tumor immune microenvironment have been identified as key factors in promoting tumor progression.^19^ Therefore, we analyzed whether *POSTN* expression influences the tumor immune microenvironment. To that end, we downloaded 2483 immune‐related genes from the ImmPort database and identified that 541 immune genes were co‐expressed with *POSTN*. Subsequent GO and KEGG analyses revealed that *POSTN* expression was correlated with cytokine‐mediated signaling pathways, MAPK and PI3K/Akt signaling pathways, positive regulation of cytokine production, and cytokine‐cytokine receptor interaction (Figure 4C). The GSEA analysis revealed that *POSTN* expression was positively correlated with chemotaxis and cell migration (Figure 4D), indicating that elevated *POSTN* expression may modulate the tumor immune microenvironment.

### 3.4 Relationship between the immune landscape of the pancreatic cancer microenvironment and *POSTN* expression

**FIGURE 5:**
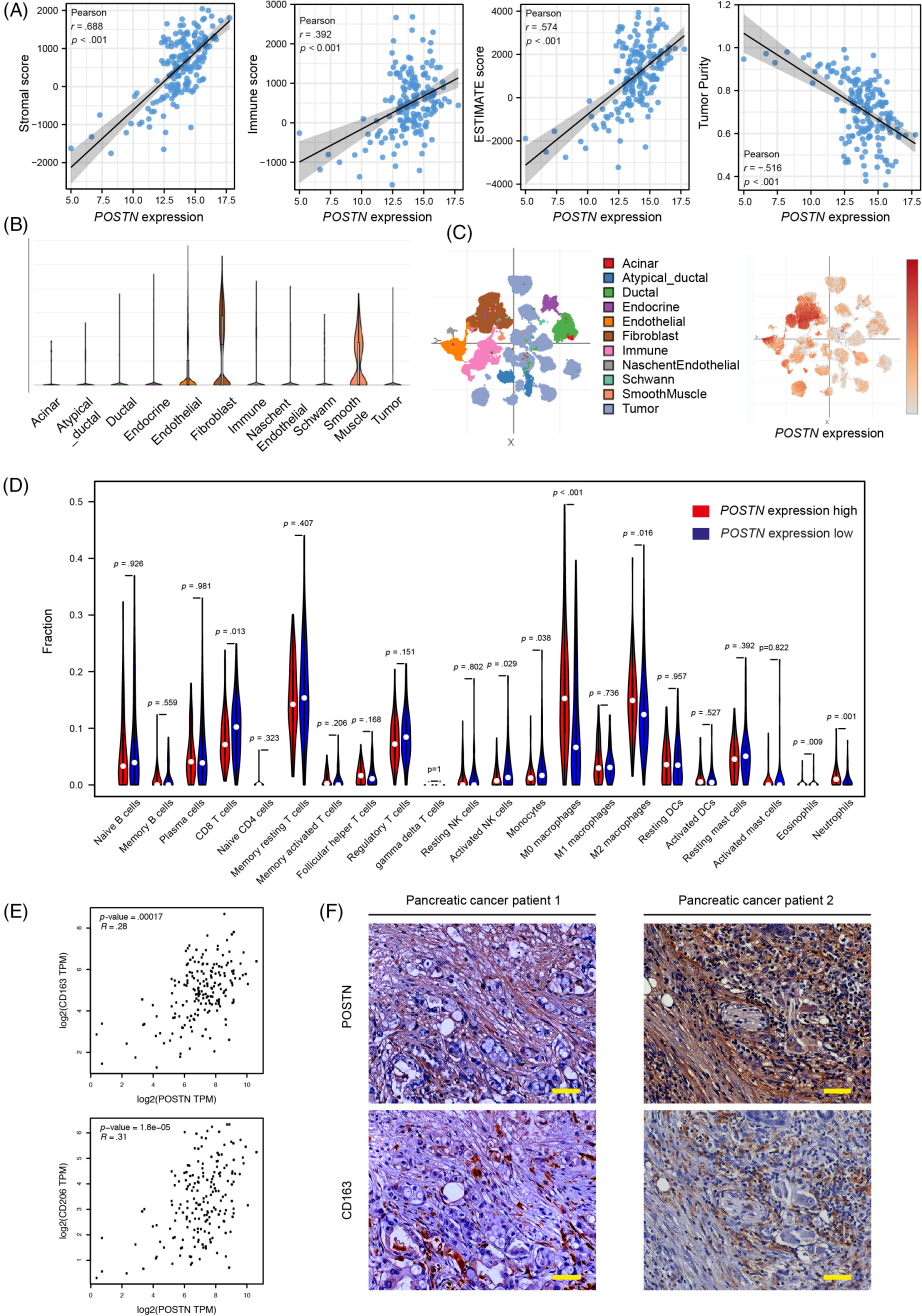
Expression of *POSTN* is associated with higher stromal infiltration. (A) Correlation between *POSTN* expression and stromal, immune, and ESTIMATE scores, as well as tumor purity, (B) violin plot for *POSTN* expression in different cell types in pancreatic cancer on the Single Cell Portal, (C) different cell types are represented by the cluster, generated from single nucleus RNA‐seq data of pancreatic cancer on the Single Cell Portal, (C) POSTN expression level is shown in different clusters, predominantly in the fibroblast cluster, (D) bar graph of immune infiltrating cells between *POSTN*‐high and‐low expression groups, (E) relationship between *POSTN* expression and M2 macrophage marker expression, and (F) Representative POSTN and CD163 staining of pancreatic cancer patient specimens (20× magnification, bar: 50 μm). TPM, transcript per million.

To explore a potential association between *POSTN* expression and the immune landscape of pancreatic cancer, we first analyzed the TCGA cohort using the ESTIMATE algorithm. We found that *POSTN* expression was positively related to stromal and immune scores (Figure 5A). Consistently, we observed a positive correlation between *POSTN* expression and ESTIMATE score, and a negative correlation with tumor purity (Figure 5A). Therefore, we next analyzed the Single Cell Portal, and single nucleus RNA‐seq data of pancreatic cancer. This analysis demonstrated that expression of *POSTN* was observed predominantly in fibroblasts and smooth muscle cells, but not in cancer cells (Figure 5B,C). These data suggest that accumulation of *POSTN*‐positive fibroblasts or CAFs are the main contributor to *POSTN* levels in bulk tissues. We further analyzed the relationship between *POSTN* expression and immune cell infiltration. Additionally, using the “CIBERSPRT” algorithm we found a positive correlation of *POSTN* expression with the infiltration levels of M0 macrophages (*p* < .001), M2 macrophages (*p* = .016), neutrophils (*p* = .001), and eosinophils (*p* = .009) (Figure 5D). In contrast, infiltration levels of CD8 T cells (*p* = .013) and activated NK cells (*p* = .029) were negatively associated with *POSTN* expression (Figure 5D). M2‐type macrophages in the tumor microenvironment contribute to tumor immune suppression.^16^ We observed that expression of *POSTN* was positively correlated with expression of the M2‐type macrophage markers *CD163* and *CD206* (Figure 5E). Furthermore, we histologically observed M2‐type macrophage marker CD163 in surgical specimens of pancreatic cancer patients (Figure 5F).

### 3.5 High expression of *POSTN* is predicted to correlate with lower sensitivity to immunotherapy using checkpoint inhibitors in pancreatic cancer

**FIGURE 6:**
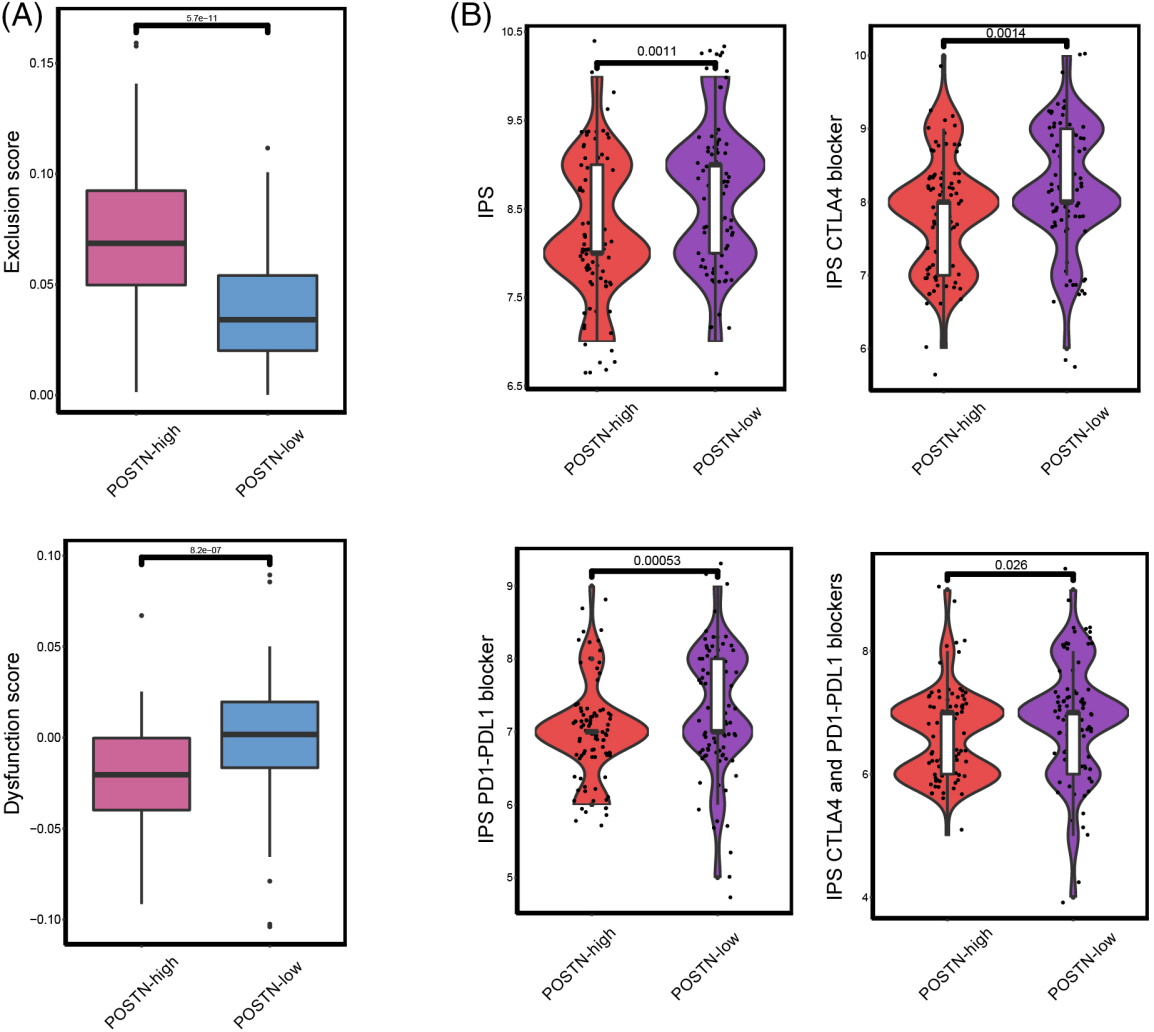
POSTN expression is associated with prediction of immune therapy efficacy. (A) Relationship between *POSTN* expression and exclusion/dysfunction scores and (B) the relationship between *POSTN* expression and immunophenoscores (IPSs).

CTLs are key immune cells that play an important role in immune function and immune surveillance.^20^ We further analyzed the relationship between *POSTN* expression and CTL exclusion and dysfunction through the TIDE score. From the TIDE algorithm, we observed a higher exclusion score (*p* < .001) and lower dysfunction score (*p* < .001) in the *POSTN*‐high expression group, indicating that there were less CTLs and less deficient CTLs in the *POSTN*‐high expression group (Figure 6A).

Next, we calculated the IPS in the *POSTN*‐high and *POSTN*‐low expression groups to determine the tumor immunogenicity for predicting response to immune checkpoint inhibitor (ICI) therapy such as CTLA‐4 and PD‐1/PDL1 inhibitors. We used the IPS dataset from the TCIA to predict the outcome of immunotherapy in pancreatic cancer patients with different *POSTN* expression status. The IPS was significantly higher in the *POSTN*‐low expression group (*p* = .001), suggesting that *POSTN* expression attenuates immunotherapy efficacy in pancreatic cancer patients. IPS scores for CTLA4 (*p* = .0014), PD‐1/PDL1 (*p* < .001), and CTLA4/PD‐1/PDL1 ICI (*p* = .026) immunotherapy were also higher in the *POSTN*‐low expression group, suggesting that lower expression of *POSTN* is associated with a better outcome with ICI treatment (Figure 6B). Taken together, our data suggest that higher *POSTN* expression is associated with higher stromal infiltration and enhanced immune‐suppressive condition in the tumor microenvironment, potentially attenuating immunotherapy efficacy in pancreatic cancer patients.

## 4 DISCUSSION

Pancreatic cancer is one of the most aggressive human malignancies with a dismal prognosis. Previous research has shown that *POSTN* expression levels may promote pancreatic cancer cell proliferation, migration, and invasion.^11^, ^12^ In the current study, we found that expression of *POSTN* is associated with shorter overall survival and recurrence‐free survival in pancreatic cancer patients (Figure 2). Further, higher expression of *POSTN* was associated with increased stromal and immune scores (Figure 5A), as well as increased infiltration of M2 macrophages (Figures 5D,6A). In addition, our data showed that higher expression of *POSTN* may contribute to low therapeutic efficacy with ICIs (Figure 6B). Taken together, higher *POSTN* expression is associated with higher stromal infiltration and enhanced immune suppression in the tumor microenvironment, and attenuates immunotherapy efficacy in pancreatic cancer patients. POSTN is predominantly expressed and secreted by activated PSCs or CAFs,^10^ suggesting that POSTN‐positive PSCs and CAFs can play an important role in remodeling the tumor immune microenvironment.

In our study, we identified *POSTN* co‐expression with immune‐related genes that are associated with cytokine‐mediated signaling pathways, positive regulation of MAPK cascade, positive regulation of cytokine production, leukocyte migration, positive regulation of cell–cell adhesion, and regulation of cell chemotaxis (Figure 4). A recent study provided evidence that POSTN plays a role in enhancing ERK and NF‐κB signaling pathways, leading to the production of cytokines that attract and mobilize macrophages. This, in turn, results in increased chemotaxis of monocytes and their polarization towards M2 macrophages.^21^ It was further suggested that POSTN in breast cancer cells promotes activation of adipose‐derived cells to become CAF‐like cells. In conclusion, POSTN‐positive cancer cells activate both CAFs and macrophages. In the case of pancreatic cancer, it has been shown that POSTN is predominantly expressed and secreted by PSCs and CAFs. Hence, it needs to be further clarified in the future whether POSTN‐positive CAFs activate signaling pathways in pancreatic cancer cells to induce infiltration and differentiation of macrophages, or POSTN‐positive pancreatic CAFs directly recruit monocyte/macrophages and support differentiation. Pancreatic CAF‐mediated monocyte/macrophage infiltration and differentiation into M2 macrophages is likely as it has been shown that CAF‐induced M2‐polarized macrophages promote hepatocellular carcinoma progression.^22^

We also identified that higher expression of *POSTN* is associated with a worse outcome with ICI treatment. Pancreatic cancer patients with low *POSTN* expression showed a better (predicted) response to CTLA4 and/or PD1 and PD‐L1 checkpoint inhibitors (Figure 6). Previous studies have demonstrated that classical ICIs have shown limited success in treating pancreatic cancer. This is primarily attributed to the immunosuppressive nature of the tumor microenvironment, immune privilege, and poor infiltration of T cells.^20^ Interestingly, in our study, we revealed that *POSTN* expression supports an immunosuppressive tumor microenvironment and reduced T cell infiltration (Figure 5). Our findings further assist in elucidating the role of POSTN in pancreatic cancer and POSTN may be a potential target to reprogram the immunosuppressive tumor microenvironment for pancreatic cancer immunotherapy. In the future, it is of interest to study whether combination treatment with POSTN inhibitors and ICIs can improve immunotherapy efficacy for pancreatic cancer patients.

## 5 CONCLUSION

Our study highlighted the significant role of stromal *POSTN* expression in pancreatic cancer. We observed that higher levels of *POSTN* were associated with shorter overall survival and recurrence‐free survival in pancreatic cancer patients. Moreover, our study indicated that elevated *POSTN* expression was correlated with increased infiltration of immune‐suppressive cells such as M2 macrophages. These findings suggested that *POSTN*‐positive CAFs play a crucial role in shaping the immune landscape of pancreatic tumors. Furthermore, we observed that higher *POSTN* expression was associated with poor response to ICIs in pancreatic cancer patients. This may be attributed to the immunosuppressive tumor microenvironment and reduced T cell infiltration by POSTN expression. Our findings suggest that targeting POSTN could potentially reprogram the immunosuppressive tumor microenvironment and enhance the efficacy of immunotherapy in pancreatic cancer. By targeting POSTN and modulating the immune tumor microenvironment, we may pave the way for more effective and personalized treatment strategies in this aggressive malignancy.

## AUTHOR CONTRIBUTIONS

**Yijun Chen:** Formal analysis (equal); investigation (equal); methodology (equal); visualization (equal); writing – original draft (equal). **Fengyu Zhang:** Formal analysis (equal); methodology (equal); writing – original draft (equal). **Bolin Zhang:** Investigation (equal). **Bogusz Trojanowicz:** Methodology (equal); resources (equal). **Monika Hämmerle:** Methodology (equal); resources (equal). **Jörg Kleeff:** Conceptualization (equal); writing – original draft (equal). **Yoshiaki Sunami:** Conceptualization (equal); formal analysis (equal); methodology (equal); visualization (equal); writing – original draft (equal).

## FUNDING INFORMATION

This work was supported in part by a research grant of the German Research Foundation [GRK 2751 InCuPanC] 449501615 to MH and JK, and China Scholarship Council (CSC) grant no. 202208080050 to YC.

## CONFLICT OF INTEREST STATEMENT

The authors have stated explicitly that there are no conflicts of interest in connection with this article.

## ETHICS STATEMENT

The study was conducted in accordance with the Declaration of Helsinki, and approved by the Ethics Committee, the institutional review board of the Medical Faculty of the Martin‐Luther‐University Halle‐Wittenberg, (Approval number: 2019‐037, on July 15, 2019).

## Data Availability

The data of this study are available from the authors upon reasonable request.
